# Analysis of prescription pattern and adverse drug reactions of drugs used in Urinary Tract Infection in reproductive age group (15-44 years) women in a Tertiary Care Hospital in Central India: An observational retrospective study

**DOI:** 10.12688/f1000research.149264.2

**Published:** 2025-02-11

**Authors:** Gayatri Patil, Sushil Varma

**Affiliations:** 1 Pharmacology, Datta Meghe Institute of Higher Education and Research, Wardha, Maharashtra, 442001, India

**Keywords:** Pattern, Prescription, urinary tract infection, adverse drug reaction, reproductive.

## Abstract

**Background:**

Urinary tract infection is considered a common bacterial infection. Nowadays there is an increase in the irrational and inappropriate use of drugs.

Adverse drug reactions have been reported to be a main cause of morbidity, hospital admission, and sometimes death. For this, it is very important to do the analysis of prescriptions and to see the adverse drug reactions due to the prescribed drugs.

**Objective:**

1. To analyze the prescribing pattern of drugs used in urinary tract infections of the reproductive age group (15-44).
2. To analyze adverse reactions due to drugs used for UTI of reproductive age group (15-44).
3. To analyze the pattern of combination therapy for urinary tract patients of reproductive age group.
4. To analyze the Fixed Drug Combination.

**Methods:**

It is a retrospective observational study. This study will include prescriptions for UTIs for females of reproductive age (15-44 years). Data will be collected from 47 prescription papers from the medical record section. Then we will review the available data for analysis of prescription patterns and adverse drug reactions.

## Introduction

Urinary tract infection occurs when microbial pathogens are present in the urinary tract. It affects both genders but it is more common in women and it affects the females of the reproductive age group (15-44 years) mostly. Another Mahadevamma et al study was done in 2011 (Aug 2011 to Dec 2011) on UTI in that they concluded that Ciprofloxacin 25(23.8%), Norfloxacin 15(14.3%), and Ceftriaxone 14(13.3%) were prescribed frequently. ^1^

In 2019 Chiang et al study was done on UTI in female patients in that study fluoroquinolone (36.4%, 16.3 million patients out of 44.9 million patients), nitrofurantoin (31.8%, 14.3 million patients out of 44.9 million patients), and trimethoprim-sulfamethoxazole (26.3%, 11.8 million patients of 44.9 million patients) this was the most prescribed drugs. ^2^

It may be because of their anatomy and reproductive physiology. Women get urinary tract infections more often because their urethra is shorter than men's and it may be due to delay in micturition, use of contraceptives, and sexual activity.

The kidneys and the ureter are involved in upper urinary tract infections, while the bladder and urethra are involved in lower urinary tract infections. ^3^

Clinically urinary tract infection is classified into – Complicated/uncomplicated and primary/recurrent. ^3^ organisms that are responsible for urinary tract infection are “Escherichia coli, Staphylococcus, Streptococcus, Klebsiella, Proteus, Corynebacterium, Neisseria and Pseudomonas species”. ^4^

Treatment of urinary tract infections may vary according to location, age of patient, and severity of presentation in the community. After the urine culture sensitivity test, empiric therapy for UTI should be initiated. The choice of medication should be determined by identifying the pathogen, assessing sensitivity through urine culture, and considering the patient's comorbid conditions. ^5^

### Treatment

Uncomplicated cystitis:

Nitrofurantoin 100mg - 5 days is the 1 ^st^ line treatment and

2 ^nd^ line treatment for uncomplicated cystitis: Trimethoprim/Sulfamethoxazole - 3 days,

oral Cephalexin 500mg - 3-7 days, or Fosfomycin 3 g -1 day.

Complicated cystitis:

Nitrofurantoin 100mg - 7 days is the 1 ^st^ line treatment and

Trimethoprim/Sulfamethoxazole -7 days, or oral Cephalexin 500 mg - 7 days, or Fosfomycin 3g every 48 hours for 3 doses is the 2nd line treatment. ^6^

Most commonly Nitrofurantoin and Sulfonamides are used in the treatment of UTI in reproductive age groups and pregnant women. ^7^

RELATED ADVERSE DRUG REACTIONS:

(1) Adverse drug reactions of Nitrofurantoin:

Nausea, loss of appetite, vomiting and diarrhea

Severe adverse reactions are:

Pulmonary toxicity (acute, subacute, chronic pulmonary reaction)

liver injuries due to nitrofurantoin

Peripheral neuropathy – is rare and it is mostly seen in patients with poor renal function and on prolonged use. ^8^

(2) Adverse drug reactions of Sulfonamides:

Sulfonamide-induced liver injury- it can be hepatocellular or cholestatic hypersensitivity reactions include fever, facial edema, lymphadenopathy, arthralgias, rash, eosinophilia, or atypical lymphocytosis (or both).

diarrhea, nausea, headaches, and dizziness, skin rash.

Rare adverse reactions:

severe allergic skin rashes, aplastic anemia, pancreatitis, serum sickness, Stevens-Johnson syndrome, agranulocytosis, nephritis, confusion seizures, and ataxia. ^9^

### Protocol

Nowadays, it is necessary to evaluate prescriptions due to the rapid changes in the prescriptions of diseases.

To analyze the irrational and inappropriate use of drugs this study is necessary.

For the improvement of patient compliance and to find out what type of adverse reactions are seen in reproductive age group female (15-44yrs) UTI patients due to prescribed drugs.

### Aim

To analyze the prescription pattern and adverse drug reactions of drugs used in urinary tract infections of reproductive age group (15-44).

### Objectives

**Primary**-

1. To analyze the prescribing pattern of drugs used in urinary tract infections of the reproductive age group (15-44).
2. To analyze adverse reactions due to drugs used for UTI of reproductive age group (15-44).

**Secondary**-

1. To analyze the pattern of combination therapy for urinary tract patients of reproductive age group.
2. To analyze the Fixed Drug Combination.

## Methods

**Study design:** Retrospective Observational Study

**Study population:** 47

**Study duration:** 18 Months (February 2024 – August 2025)

### Inclusion criteria

The study will include prescriptions of UTI for Females of the reproductive age group (15-44 years).

### Exclusion criteria

ICU patients

Incomplete case files.

### Study setting

This research study will be carried out in the Department of Pharmacology of Rural Tertiary Care Hospital in Central India.

### Sample size estimation

Daniel Formula:

Z = statistic for a level of confidence =1.96

P = Prevalence of urinary tract infection =3.14 = 0.0314 [prevalence taken as per the reference article Mavi A, Rathi I, Shannawaz M, Saeed S, Hasan S. Correlates of Urinary Tract Infections Among Women of Reproductive Age in India: A Systematic Review. Cureus. 2024 Apr;16(4)]

d = Desired error of margin = 5% = 0.05

n = Z ^2^ × P (1 − P)/d ^2^

n = (1.96) ^2^ × 0.0314 × [1 − 0.0314]/(0.05) ^2^ = i.e. 47 patients needed in the study

**Statistical methods**: Student's t-test, Chi-square Test.

**Software used**: SPSS 27.0 version.

**Study design**: Retrospective observational study.

### Data collection and procedure

We will collect recorded files of patients of reproductive age group female (15-44yrs) UTI patients from the medical record section. And Analyze prescriptions and adverse drug reactions of patients. Table 1 details the parameters included in the study. Data will be analyzed using a suitable Statistical Test SPSS- 20.

**Table 1.** Demographic and drug details of patients.

NAME	AGE/SEX	MRD NO.	OPD NO.	DIAGNOSIS	DRUGS USED	DOSE OF THE DRUG	ANY IRRATIONAL IN PRESCRIPTION	DURATION OF DRUG THERAPY	ADVERSE DRUG REACTIONS	DURATION OF ILLNESS	COMORBIDITIES	PREGNANCY STATUS	RECURRENT INFECTIONS
												

### Dissemination

The findings of this study will be used for metanalysis.

### Study status

The study is yet to be started.

## Discussion

Nowadays irrational and inappropriate use of drugs is a major concern. Irrational drug use may cause adverse consequences including drug resistance and adverse drug reactions. ^10^^,^^11^ Nitrofurantoin, Trimethoprim-Sulfamethoxazole, and Fluoroquinolones are the most commonly used medications for UTI. ^12^ In our study we will analyze the prescriptions of drugs that will be prescribed for UTI.

For the improvement of patient compliance, it is very important to analyze prescription patterns and adverse drug reactions (ADRs). The benefits of our research will be patient care improvement and risk assessment and lesser events of adverse drug events associated with the prescribed drugs. Also, we will be analyzing the rationality of prescribing FDC and combination therapy. As well as their overall impact on the outcome of treatment.

This analysis is for the use of evidence-based medicine in tertiary care centers, to fulfill the drug information needs of the physician and also for improvement in the quality of healthcare by giving feedback to prescribers. This is an observational, retrospective study that will aim at the analysis of drug prescription patterns and adverse drug reactions due to drug prescription in urinary tract infections at tertiary health care centers.

### Ethical considerations

1. Ethical Committee approval is obtained.

Consent to participate:

As it is a retrospective there is no requirement from our institutional Ethical committee for getting the data of the past patient it is waived off for this study, Also we have taken proper permission and a letter from the concerned faculty of the Hospital (CMS) from the medical record section for the previous data.

## Data Availability

No data are associated with this article.
