# Analysis of prescription pattern and adverse drug reactions of drugs used in Urinary Tract Infection in reproductive age group (15-44 years) women in a Tertiary Care Hospital in Central India: An observational retrospective study

**DOI:** 10.12688/f1000research.149264.5

**Published:** 2025-09-12

**Authors:** Gayatri Patil, Sushil Varma

**Affiliations:** 1 Pharmacology, Datta Meghe Institute of Higher Education and Research, Wardha, Maharashtra, 442001, India

**Keywords:** Pattern, Prescription, urinary tract infection, adverse drug reaction, reproductive.

## Abstract

**Background:**

Urinary tract infection (UTI) is a frequent bacterial infection among women of reproductive age. Inappropriate and irrational prescribing of antibiotics **—such as the use of unapproved fixed-dose combinations or empirical broad-spectrum therapy—**has contributed to growing antimicrobial resistance. Adverse drug reactions (ADRs) further complicate treatment outcomes, yet remain underreported, especially in routine outpatient practice. Therefore, an assessment of current prescribing patterns is essential to promote rational drug use. Additionally, evaluation of adverse drug reactions remains important for patient safety and pharmacovigilance.

**Objectives:**

• To describe the prescribing patterns of drugs used for urinary tract infections (UTIs) among women of reproductive age (15–44 years).
• To evaluate the incidence, type, and severity of adverse drug reactions (ADRs) as documented in hospital records.
• To assess the prescription of fixed-dose combinations (FDCs) and combination antimicrobial therapy in relation to standard treatment guidelines (WHO, NLEM 2022).

**Methods:**

This is a retrospective observational study based on outpatient department (OPD) prescriptions for women aged 15–44 years diagnosed with community-acquired, uncomplicated UTIs at a tertiary care hospital in Central India. Data from 139 prescription records **will be retrieved from the medical record section and analyzed.** Prescribing indicators, documented ADRs, and use of FDCs will be assessed against national guidelines using descriptive statistics, chi-square tests, and logistic regression analysis.

## Introduction

Urinary tract infection (UTI) is the most prevalent bacterial infection among women of reproductive age (15–44 years) because of their anatomical and physiological properties like shorter urethra, sexual activity, contraceptive use, and delayed micturition. ^1^ ^,^ ^2^

UTIs are symptomatically differentiated into uncomplicated or complicated, and primary or recurrent, the most common uropathogen being Escherichia coli followed by Klebsiella, Staphylococcus, Streptococcus, and Pseudomonas species. ^3^

A Karnataka, India, study conducted by Mahadevamma and Sarala (2011) revealed ciprofloxacin (23.8%), norfloxacin (14.3%), and ceftriaxone (13.3%) to be frequently prescribed in patients with UTI. ^4^ Likewise, Chiang et al. (2019) indicated that fluoroquinolones (36.4%), nitrofurantoin (31.8%), and trimethoprim-sulfamethoxazole (26.3%) were the most frequently prescribed drugs for UTIs among women in a US-based review of 44.9 million prescriptions. ^5^

Antibiotic resistance increases, especially to fluoroquinolones and third-generation cephalosporins, in Indian environments, ^6^ ^,^ ^7^ leading to a change to nitrofurantoin and sulfonamides as first-line therapy.

Although ADRs are an important concern in antimicrobial treatment, they remain underreported in retrospective hospital records, particularly in resource-poor environments. Nitrofurantoin is known to cause gastrointestinal disturbance, pulmonary toxicity, and liver damage. ^8^ Sulfonamides are responsible for hypersensitivity reactions, hepatotoxicity, Stevens–Johnson syndrome, and hematologic toxicity. ^9^

In spite of the availability of guidelines like the National List of Essential Medicines (NLEM 2022) and WHO procedures, irrational prescribing continues in the form of long-term use of broad-spectrum antibiotics and irrational fixed-dose combinations (FDCs). ^10^

There is an obvious lack of Indian data on prescribing patterns and ADRs in reproductive-age women specifically.

### Protocol

Nowadays, it is necessary to evaluate prescriptions due to the rapid changes in the prescriptions of diseases.

To analyze the irrational and inappropriate use of drugs this study is necessary.

For the improvement of patient compliance and to find out what type of adverse reactions are seen in reproductive age group female (15-44yrs) UTI patients due to prescribed drugs.

## Aim

To analyze the prescription pattern and adverse drug reactions of drugs used in urinary tract infections of reproductive age group (15-44).

## Objectives

• To describe the prescribing patterns of drugs used for urinary tract infections (UTIs) among women of reproductive age (15–44 years).
• To evaluate the incidence, type, and severity of adverse drug reactions (ADRs) as documented in hospital records.
• To assess the prescription of fixed-dose combinations (FDCs) relevant to UTI management (e.g., Co-trimoxazole, Amoxicillin–Clavulanate) and their adherence to standard treatment guidelines (WHO, NLEM 2022).

## Methods

### Population and case definition

The study included prescription records of female patients aged 15–44 years diagnosed with community-acquired, uncomplicated UTI by a physician.

Diagnosis of community-acquired uncomplicated UTI was based on documented clinical symptoms (dysuria, urgency, frequency, suprapubic pain), with or without supporting urine analysis or culture reports, as recorded by treating physicians.

### Inclusion criteria

Outpatient records of women aged 15–44 years with a diagnosis of community-acquired uncomplicated UTI.

### Exclusion criteria

• Inpatient, ICU, or hospital-acquired UTI cases.
• Complicated UTI cases (e.g., with renal involvement, pregnancy, catheterization).
• Incomplete case records.

### Study setting

This is a **retrospective observational study** conducted at a rural tertiary care hospital in Central India. Data were collected exclusively from outpatient records. **No telephonic follow-up or direct patient contact was performed.**

### Sample size estimation

Calculated using a 10% ADR prevalence assumption → 139 records.

Subgroup analyses may be underpowered; however, the study is adequately powered for primary objectives.

While the sample size was estimated using an assumed 10% ADR prevalence, it is primarily adequate for the main objective of analyzing prescribing patterns. ADR analysis will remain exploratory due to the limitations of retrospective data and possible under-reporting.

Using the **Daniel formula**:

$$n=Z^{2}\times P(1-P)/d^{2}n=Z^{2}\times P(1-P)/d^{2}n=Z^{2}\times P(1-P)/d^{2}$$

Where:

• Z = 1.96 for 95% confidence
• P = 0.10 (assumed 10% prevalence of ADRs based on prior studies e.g., Fatima et al., 2015)
• d = 0.05d = 0.05 (desired precision = 5%)

$n={\left(1.96\right)}^{2}\times 0.10\times \left(1-0.10\right)/{\left(0.05\right)}^{2}=138.3\approx 139\;\text{patients}$

Final sample size: 139 prescription records

### Study design

This is a **retrospective observational study** using **OPD records** of women with UTI in the reproductive age group.

No patient contact or follow-up will be conducted.

No data will be taken from inpatients, ICU, or hospital-acquired infections.

### Statistical methods

Data will be analyzed using **SPSS version 27.0**.

• Descriptive statistics: Means ± SD for continuous variables, frequencies (%) for categorical variables.
• Chi-square test: To assess association between drug class and ADR occurrence.
• Logistic regression: To evaluate predictors of ADRs (e.g., age, comorbidity, drug class).
• Records with missing variables will be excluded from respective analyses. A *p* value <0.05 will be considered statistically significant.

### Software used

All statistical analyses will be performed using **SPSS Version 27.0 (IBM Inc., USA).**

### Data collection and procedure

We will collect recorded files of patients of reproductive age group female (15-44yrs) UTI patients from the medical record section. And Analyze prescriptions and adverse drug reactions of patients. Table 1 details the parameters included in the study. Prescriptions were reviewed for demographics, diagnosis, prescribed drugs, comorbidities, FDCs, and adverse drug reactions (ADRs), if documented by the treating physician. Only ADRs recorded in OPD case files will be analyzed, and we recognize that minor or non-serious ADRs are likely underreported.

**Table 1.** Parameters and operational definitions used in data collection.

Parameter	Operational definition
Age/Sex	Age in completed years; sex as recorded (female only included)
MRD No. /OPD No.	Unique identifiers from the hospital medical records
Diagnosis	Community-acquired uncomplicated UTI diagnosed by the treating physician, based on documented clinical symptoms (dysuria, urgency, frequency, suprapubic pain), with or without supporting urine analysis or culture reports
Drugs Used	All antimicrobial and supportive drugs prescribed
Dose & Duration	As per prescription record (in mg/day and number of days)
Irrational Prescription	Any prescription deviating from WHO prescribing indicators or National List of Essential Medicines (NLEM) guidelines
Combination Therapy	Use of two or more antimicrobials prescribed for the same UTI episode
Fixed Drug Combinations	Pharmaceutical formulations with ≥2 active ingredients, e.g., Co-trimoxazole, Amoxicillin-Clavulanate; non-standard combinations (e.g., Nitrofurantoin + B6) excluded
Adverse Drug Reactions (ADR)	Any noxious, unintended medical occurrence temporally associated with drug use, **as documented in OPD records by the treating physician**, classified using WHO-UMC criteria. Minor/non-serious ADRs may be underreported due to retrospective design
Comorbidities	Presence of chronic condition (e.g., diabetes, hypertension) in the patient record
Pregnancy Status	As per documented clinical status during treatment
Recurrent UTI	≥2 episodes within 6 months or ≥3 episodes within 12 months
Duration of Illness	Duration between onset of symptoms and resolution as noted in case file

### Statistical analysis

Data will be entered and analyzed using SPSS version 27.0. Descriptive statistics will be used to summarize baseline characteristics such as age, drug type, and comorbidities (mean ± SD or frequencies as appropriate).

Chi-square test will be used to examine associations between drug class and adverse drug reactions.

If the sample permits, binary logistic regression analysis will be performed to identify predictors of ADRs (e.g., age, pregnancy status, polypharmacy, comorbidity).

Missing data will be excluded from inferential analysis, and the extent of missingness will be reported.

A p-value < 0.05 will be considered statistically significant.

### Dissemination

The results of this study may contribute to future systematic reviews or meta-analyses focused on prescribing patterns and adverse drug reactions in urinary tract infections.

### Study status

The study is yet to be started.

## Discussion

Today irrational and in appropriate use of drugs is a serious issue. Irrational drug use can lead to adverse effects such as drug resistance and drug side effects. ^11^ ^,^ ^12^ Nitrofurantoin, Trimethoprim-Sulfamethoxazole, and Fluoroquinolones are the drugs most frequently used to treat UTI. ^13^ In this study we will examine the prescriptions of the drugs that will be prescribed for UTI.

This research will close that gap by assessing actual prescribing practice and pharmacovigilance in outpatient UTI care. This research is anticipated to underscore irrational prescribing and under-reporting of ADRs in the care of UTIs, which lead to antibiotic resistance and suboptimal patient outcomes. ^6^ ^,^ ^7^

The earlier research has reported poor compliance with guidelines and excessive use of combination therapy in Indian practice. ^4^ ^,^ ^10^ Lack of systematic monitoring of ADR can further hamper rational use of drugs, particularly among rural patients who have limited diagnostic facility access. ^8^ ^,^ ^9^

By analyzing prescription audit and ADR patterns, this paper will help support local antimicrobial stewardship and inform future policy.

For the improvement of patient compliance, it is very important to analyze prescription patterns and adverse drug reactions (ADRs). The benefits of our research will be patient care improvement and risk assessment and lesser events of adverse drug events associated with the prescribed drugs. Also, we will be analyzing the rationality of prescribing FDC and combination therapy. As well as their overall impact on the outcome of treatment.

This analysis is for the use of evidence-based medicine in tertiary care centers, to fulfill the drug information needs of the physician and also for improvement in the quality of healthcare by giving feedback to prescribers. This is an observational, retrospective study that will aim at the analysis of drug prescription patterns and adverse drug reactions due to drug prescription in urinary tract infections at tertiary health care centers.

### Limitations

This study has certain limitations. As a retrospective record-based study, under-reporting of ADRs in OPD records is a major concern, as minor or non-serious reactions are less likely to be documented. Diagnostic variability, arising from reliance on physician-recorded symptoms with or without laboratory confirmation, may also affect case ascertainment. Furthermore, the sample size may be insufficient for detailed subgroup analyses. Prospective studies are better suited to systematically capture ADRs and provide stronger evidence.

### Ethical considerations

The study was approved by the **Institutional Ethics Committee of Datta Meghe Institute of Higher Education and Research (Deemed to be University)** (Reference No: DMIHER (DU)/IEC/2024/159).

As this was a retrospective record-based study, **informed consent was waived**. Institutional permission was obtained from the **Chief Medical Superintendent (CMS)** and the Medical Records Section to access anonymized.

## Data availability

No data are associated with this article at this stage. Data will be made available upon completion of the study, subject to institutional approval and journal policy.
