# Supplementary material for: Analysis of prescription pattern and adverse drug reactions of drugs used in Urinary Tract Infection in reproductive age group (15-44 years) women in a Tertiary Care Hospital in Central India: An observational retrospective study
Source: F1000Res. 2025 Sep 12;13:417. Originally published 2024 Apr 29. [Version 5] doi: 10.12688/f1000research.149264.5 (PMC12441663; doi:10.12688/f1000research.149264.5)
Supplement: Peer review history

# Reviewer report: Reviewer response for version 5

**DOI:** 10.5256/f1000research.188018.r413764

**Published:** 2025-09-16

**Authors:** Abayeneh Girma

**Affiliations:** 1 Mekdela Amba University, Tulu Awlya, South Wollo Zone, Ethiopia

General comments

 This protocol describes a retrospective observational study aiming to analyze prescribing patterns and documented adverse drug reactions (ADRs) for urinary tract infections (UTIs) in women of reproductive age in Central India. The topic is highly relevant to antimicrobial stewardship and pharmacovigilance in a resource-limited setting. While the authors have made revisions through several versions, significant methodological and conceptual concerns remain, particularly regarding the feasibility of the ADR objective and the statistical plan. The study design is appropriate for analyzing prescription patterns but is fundamentally flawed for its secondary aim of analyzing ADRs.

**1. Introduction: **The rationale for focusing specifically on the reproductive age group (15-44 years) is not strongly justified. While anatomical factors are mentioned, a clearer link to unique prescribing challenges or ADR profiles in this group (e.g., teratogenicity concerns, interaction with contraceptives) would strengthen the argument. Again, some references are quite old (e.g., Roujeau 1995, Foxman 2002, and more than 2 studies conducted in 2008). Incorporating more recent literature on Indian prescribing patterns and resistance trends would better contextualize the study.

**2. Methods, particularly on statistical analysis:** The description is insufficient for replication. There is no detail on which specific covariates will be included in the logistic regression model to "identify predictors of ADRs." How missing data will be handled (beyond exclusion). How model assumptions will be checked.

Is the study design appropriate for the research question?

No

Is the rationale for, and objectives of, the study clearly described?

No

Are sufficient details of the methods provided to allow replication by others?

No

Are the datasets clearly presented in a useable and accessible format?

No

Reviewer Expertise:

Microbiology

I confirm that I have read this submission and believe that I have an appropriate level of expertise to confirm that it is of an acceptable scientific standard, however I have significant reservations, as outlined above.

# Reviewer report: Reviewer response for version 4

**DOI:** 10.5256/f1000research.187324.r410764

**Published:** 2025-09-09

**Authors:** Abayeneh Girma

**Affiliations:** 1 Mekdela Amba University, Tulu Awlya, South Wollo Zone, Ethiopia

General comments

 This manuscript describes a protocol for a retrospective observational study aimed at analyzing prescription patterns and adverse drug reactions (ADRs) related to urinary tract infection (UTI) treatment in reproductive-age women in Central India. While the topic is relevant and timely given the global concern about antimicrobial resistance and rational drug use, the manuscript suffers from significant methodological and conceptual flaws that undermine its scientific rigor, feasibility, and potential impact. Despite multiple revisions, key issues remain unaddressed.

1. **Study design and methodology:** The study is described as retrospective, yet earlier versions mentioned telephonic follow-up for ADR data—a prospective element. Although the authors now claim to have removed this, the protocol still lacks clarity regarding how ADRs will be accurately captured from retrospective records, which are notoriously unreliable for ADR reporting. Again, the diagnostic criteria for UTI are not clearly defined. The authors state that diagnosis is based on “documented clinical symptoms with or without urine culture,” but this is vague and may lead to inclusion of misdiagnosed cases, affecting the validity of prescription pattern analysis. Moreover, the sample size (n=139) is now based on an assumed 10% ADR prevalence, which is an improvement. However, this may still be insufficient for subgroup analyses (e.g., by drug class, comorbidity), and no power calculation is provided for these secondary outcomes.
2. **Data collection and ADR assessment:** Relying solely on hospital records for ADR data is a major limitation, as ADRs are widely underreported in such sources. The authors do not propose any method to mitigate this bias (e.g., standardized chart review tools, trigger methods). Again, the parameters listed in Table 1 lack operational definitions. For example, “irrational prescription” is not defined according to any specific guideline (e.g., WHO/INRUD, NLEM). Similarly, “combination therapy” and “fixed-dose combinations” are poorly operationalized.
3. **Statistical analysis:** The statistical plan is superficial. While chi-square and logistic regression are mentioned, there is no detail on how covariates (e.g., age, pregnancy, comorbidity) will be handled, how missing data will be managed, or how model assumptions will be checked. Again, the plan to exclude missing data from inferential analysis may introduce bias if data are not missing completely at random.
4. **Ethical and practical issues:** The waiver of consent for retrospective data collection is appropriate, but the authors do not address how patient confidentiality will be maintained during data handling and analysis. Again, the study setting (“a rural tertiary care hospital in Central India”) is described, but the generalizability of findings to other settings (e.g., urban, primary care) is limited and not discussed.
5. **Clarity and reproducibility:** The manuscript contains inconsistencies and redundancies, particularly in the Introduction and Discussion. The rationale for focusing solely on reproductive-age women is not sufficiently justified. Again, the protocol does not provide enough detail for replication. For example, the process of prescription review, criteria for irrationality, and ADR classification (WHO-UMC) are mentioned but not elaborated.
6. **Finally, I have some minor issues. ** (i) The abstract remains brief and lacks specific details about methods and expected outcomes. (ii) The Introduction cites outdated references (e.g., from 2002, 2008) and does not adequately contextualize the study within current Indian or global AMR stewardship efforts. (iii) The Discussion is generic and does not anticipate potential limitations or challenges (e.g., missing data, diagnostic variability).

Is the study design appropriate for the research question?

No

Is the rationale for, and objectives of, the study clearly described?

No

Are sufficient details of the methods provided to allow replication by others?

No

Are the datasets clearly presented in a useable and accessible format?

No

Reviewer Expertise:

Microbiology

I confirm that I have read this submission and believe that I have an appropriate level of expertise to state that I do not consider it to be of an acceptable scientific standard, for reasons outlined above.

# Response

**Published:** 2025-09-10

**Authors:** DR. GAYATRI PATIL

**Affiliations:** PHARMACOLGY, Not Applicable, WARDHA, MAHARASHTRA, India

We thank the reviewer for the thoughtful feedback and valuable suggestions. We acknowledge the limitations raised, particularly regarding the retrospective design and the challenges of ADR capture from medical records. However, our study is based on an IEC-approved retrospective protocol, and therefore we are unable to incorporate prospective elements.

 We agree that ADR under-reporting, possible diagnostic variability, and limited generalizability are important limitations, and these have been acknowledged in the manuscript. Our primary objective remains the analysis of prescribing patterns, with ADR analysis kept exploratory within the constraints of available data.

 We appreciate the reviewer’s insights, which have helped us to better frame the scope and limitations of our study.

# Reviewer report: Reviewer response for version 4

**DOI:** 10.5256/f1000research.187324.r411054

**Published:** 2025-09-09

**Authors:** Sandip Mukhopadhyay

**Affiliations:** 1 Epidemiology - Pharmacology, ICMR-National Institute for Research in Bacterial Infections, Kolkata, West Bengal, India

The author had chosen an important topic to explore. Urinary tract infection is an important cause of morbidity among the patients. Use of antimicrobial may be monitored through prescription audit-like activities to detect rationality of prescription.

 However, a number of issues need attention:

 1. Rationale: Promoting rational use of drug is primarily 5Rs: right drug, right dose, right patient, right route and right time. Where checking prescription pattern partly associates with Rational use of drug, ADR may occur even with rational use (e.g. type A reactions in many occasions, Bizarre/ type B reactions, type C reactions, delayed reactions/ type D reactions, end of dose/ type E reactions). Hence, the line in the abstract section **"Therefore, an assessment of current prescribing patterns and ADRs is essential to promote rational drug use" **may be re-phrased after removing the ADR from the sentence. 

  2. The objectives 1 & 3 are feasible through a cross-sectional study design/ from record based studies and justifiable as it will be less resource intensive and faster. 

 3. Collecting retrospective Adverse drug reaction data from OPD prescriptions : retrospective data collection for such objective will lead to bias in the data. There is always possibility of high number of missing data due to under-reporting of minor/ non-serious ADRs, moving to other health facilities if there is ADR and even due to lack of follow ups.

 This could be solved only through prospective study designs. Otherwise, this objective may be dropped.

 4. Operational definition of uncomplicated UTI seemed a bit abstract. It is only based only on clinical symptoms. Ideally, it should include urinalysis and microbiological examination. 

 5. Sample size estimation should be based on primary objective (may be the objective no 1), then the question of prevalence of ADR should come.

 6. In the protocol, the statistics part should come at the last part/ end of the method section

Is the study design appropriate for the research question?

Partly

Is the rationale for, and objectives of, the study clearly described?

Partly

Are sufficient details of the methods provided to allow replication by others?

Partly

Are the datasets clearly presented in a useable and accessible format?

Not applicable

Reviewer Expertise:

Antimicrobial resistance, Clinical trials & other clinical researches, population based studies.

I confirm that I have read this submission and believe that I have an appropriate level of expertise to confirm that it is of an acceptable scientific standard, however I have significant reservations, as outlined above.

# Response

**Published:** 2025-09-10

**Authors:** DR. GAYATRI PATIL

**Affiliations:** PHARMACOLGY, Not Applicable, WARDHA, MAHARASHTRA, India

We sincerely thank the reviewer for the constructive and valuable comments. We have carefully revised the manuscript to address each point as detailed below.

**Comment 1:**

 Therefore, an assessment of current prescribing patterns and ADRs is essential to promote rational drug use may be re-phrased after removing the ADR from the sentence.

**Response:**

 We agree. The abstract has been revised to:

 Therefore, an assessment of current prescribing patterns is essential to promote rational drug use. Additionally, evaluation of adverse drug reactions remains important for patient safety and pharmacovigilance.

**Comment 2:**

 Objectives 1 & 3 are feasible through a cross-sectional/record-based design. Objective 2 on ADRs is less feasible in this design.

**Response:**

 We acknowledge this point. Objectives 1 (prescription patterns) and 3 (FDC adherence) are indeed fully aligned with a retrospective design. Objective 2 has been clarified as:

 To evaluate the incidence, type, and severity of adverse drug reactions (ADRs) **as documented in hospital records**.

 This limits ADR assessment only to available records.

**Comment 3:**

 Retrospective ADR data collection may lead to bias and missing data; this objective is best suited for prospective studies.

**Response:**

 We agree. We have emphasized in the **Methods** that only physician-documented ADRs will be captured from OPD records, and in the **Discussion,** we explicitly highlight the limitation of under-reporting in retrospective data. The ADR objective will therefore remain exploratory.

**Comment 4:**

 Operational definition of uncomplicated UTI is too abstract; should include urinalysis and microbiology.

**Response:**

 We thank the reviewer. The definition has been revised to:

 Diagnosis of community-acquired uncomplicated UTI was based on documented clinical symptoms (e.g., dysuria, urgency, frequency, suprapubic pain), with or without supporting urine analysis or culture reports.

 Table 1 has been updated accordingly.

**Comment 5:**

 Sample size estimation should be based on the primary objective (prescription pattern), not ADR prevalence.

**Response:**

 We acknowledge this. While the calculation was based on an assumed ADR prevalence, we have clarified in the **Sample Size section** that the sample is primarily adequate for the primary objective (prescription pattern analysis). ADR analysis is exploratory.

**Comment 6:**

 Statistics section should come at the end of Methods.

**Response:**

 Accepted. The **Statistical Analysis** subsection has been moved to the last part of the Methods for improved logical flow.

 We thank the reviewer once again for the valuable feedback, which has helped us to improve the clarity and scientific rigor of our manuscript.

# Reviewer report: Reviewer response for version 3

**DOI:** 10.5256/f1000research.185974.r402772

**Published:** 2025-08-26

**Authors:** Abayeneh Girma

**Affiliations:** 1 Mekdela Amba University, Tulu Awlya, South Wollo Zone, Ethiopia

**General comments**

 The manuscript addresses an important topic—prescription patterns and adverse drug reactions (ADRs) in urinary tract infections (UTIs) among reproductive-age women in Central India. However, despite revisions, significant methodological and conceptual flaws persist, compromising the study's scientific rigor, reproducibility, and potential impact. The study design, sample size justification, and clarity of objectives remain inadequate, as highlighted by previous reviewers. Below are the specific concerns that warrant rejection.

**1. Study design and methodology:**

 (i) Retrospective vs. prospective contradictions: The authors claim a retrospective design but propose telephonic follow-ups for ADR data, introducing a prospective element. This hybrid approach lacks justification and risks bias (e.g., recall bias, non-response bias).

 (ii) Data source clarity: The manuscript does not clearly specify whether UTIs are community-acquired or hospital-acquired, nor does it define diagnostic criteria (e.g., symptoms, culture results). This omission limits the interpretability of prescription patterns.

 (iii) ADR collection: Relying on hospital records for ADRs is problematic due to underreporting. The plan to contact patients by phone is not standardized or ethically clarified (e.g., consent process).

**2. Sample size and statistical power: **The revised sample size calculation (n=139) is based on an assumed 10% prevalence of ADRs, which is an improvement. However, the justification remains weak, and the sample size may still be insufficient for robust conclusions, especially for secondary outcomes like combination therapy analysis. Again, the study lacks power calculations for subgroup analyses (e.g., by drug class, age, or comorbidity), which are critical for meaningful inferences.

**3. Objectives and scope:** The primary objectives are overly broad. For example, "analyzing adverse reactions" lacks specificity (e.g., which ADRs, severity grading). Additionally, the secondary objective to analyze fixed-dose combinations (FDCs) is commendable but poorly operationalized. Examples like "Nitrofurantoin + Vitamin B6" are questionable, as Vitamin B6 is not a standard UTI treatment.

**4. Ethical and practical issues: **While ethical approval is mentioned, the waiver for patient consent in retrospective data collection is noted. However, telephonic follow-ups for ADRs would require explicit ethical approval and consent, which is not discussed. In addition, the study setting (rural tertiary care hospital) is described, but the representativeness of the sample for broader populations (e.g., urban or primary care settings) is unclear.

**5. Peer review responses:** The authors’ responses to reviewers (e.g., sample size recalculation, ADR methodology) are superficial. Key critiques (e.g., hospital vs. community UTI distinction, statistical methods) remain unaddressed. Moreover, the introduction and discussion sections lack critical synthesis of prior literature (e.g., regional antibiotic resistance trends in Central India).

**6. Statistical analysis: **The description of statistical methods is insufficient. For example: No details on covariates, regression models, or handling missing data. Again, the plan to use chi-square tests and logistic regression is mentioned but not elaborated (e.g., how predictors like age or comorbidity will be incorporated).

**7. Clarity and reproducibility:** Table 1 (Data Collection) lists parameters but lacks operational definitions (e.g., "irrational prescription" criteria). In addition, the study's retrospective nature limits its ability to capture ADRs accurately, as these are often underreported in hospital records.

Is the study design appropriate for the research question?

No

Is the rationale for, and objectives of, the study clearly described?

No

Are sufficient details of the methods provided to allow replication by others?

No

Are the datasets clearly presented in a useable and accessible format?

No

Reviewer Expertise:

Microbiology

I confirm that I have read this submission and believe that I have an appropriate level of expertise to state that I do not consider it to be of an acceptable scientific standard, for reasons outlined above.

# Response

**Published:** 2025-08-26

**Authors:** DR. GAYATRI PATIL

**Affiliations:** PHARMACOLGY, Not Applicable, WARDHA, MAHARASHTRA, India

Dear Reviewer,

 We sincerely thank you for your thoughtful review and constructive feedback. We carefully revised the manuscript to address your concerns while maintaining the retrospective design already approved by the Institutional Ethics Committee. Below, we outline our responses and the corresponding changes made:

**1. Study design and methodology**

- **Comment:** Contradiction between retrospective design and telephonic follow-up; unclear community vs. hospital-acquired cases; missing diagnostic criteria.
- **Response:** We have clarified that the study is a **retrospective observational record-based study only**, with **no telephonic follow-up or direct patient contact** (Methods, Study Design).
  We also explicitly state that included cases are **community-acquired, uncomplicated UTIs** diagnosed by **clinical symptoms ± urine culture** (Methods, Population and Case Definition).

**2. Sample size and statistical power**

- **Comment:** Justification remains weak; subgroup analyses underpowered.
- **Response:** The **ADR prevalence-based calculation (n=139)** has been retained. We added a statement that while subgroup analyses may be underpowered, the sample is adequate for the **primary objectives** (Methods, Sample Size).

**3. Objectives and scope**

- **Comment:** Objectives too broad; unclear ADR and FDC evaluation.
- **Response:** Objectives have been **refined for precision**:
  - ADRs now specified as “ **incidence, type, and documented severity**.”
  - FDC evaluation restricted to **standard, guideline-recommended combinations (e.g., Co-trimoxazole, Amoxiclav)** (Objectives section).

**4. Ethical and practical issues**

- **Comment:** Waiver of consent unclear; telephonic follow-up ethics not addressed.
- **Response:** We clarified that **consent was waived by the IEC** since this is retrospective, with **no patient contact**. Institutional permission was obtained from the **Chief Medical Superintendent and Medical Records Section** (Ethics section).

**5. Statistical analysis**

- **Comment:** Methods insufficient; no details on regression or handling missing data.
- **Response:** The **Statistical Analysis** section has been expanded:
  - Descriptive statistics for baseline data.
  - **Chi-square** for association between drug class and ADRs.
  - **Logistic regression** for predictors (age, comorbidity, drug class).
  - Handling of missing data specified.
  - SPSS v27 mentioned.

**6. Clarity and reproducibility (Table 1)**

- **Comment:** Operational definitions missing.
- **Response:** We revised **Table 1** to include clear operational definitions for irrational prescription, recurrent UTI, ADR (WHO-UMC), and comorbidity.

**7. Introduction and Discussion**

- **Comment:** Lack of regional resistance data and critical synthesis.
- **Response:**  We acknowledge the reviewer’s suggestion regarding inclusion of regional resistance data and critical synthesis. Our original Introduction and Discussion were drafted to maintain brevity and focus on prescribing trends and ADRs. While we did not insert new sentences in the main text, we have carefully reviewed relevant Central India resistance reports (e.g., Taneja & Sharma, 2019; Dutta et al., 2023) and confirm that our findings are consistent with these patterns. To preserve flow and word limits, these data have been emphasized in our response here rather than in the manuscript body.

# Reviewer report: Reviewer response for version 3

**DOI:** 10.5256/f1000research.185974.r402774

**Published:** 2025-08-13

**Authors:** Niels Frimodt-Møller

**Affiliations:** 1 Copenhagen University Hospital, Copenhagen, Denmark

The authors have responded adequately to my comments, no further comments.

Is the study design appropriate for the research question?

No

Is the rationale for, and objectives of, the study clearly described?

No

Are sufficient details of the methods provided to allow replication by others?

No

Are the datasets clearly presented in a useable and accessible format?

No

Reviewer Expertise:

Clinical microbiology, urinary tract infections, E.coli pathogenesis, antibiotic activity in vitro and in vivo

I confirm that I have read this submission and believe that I have an appropriate level of expertise to confirm that it is of an acceptable scientific standard.

# Reviewer report: Reviewer response for version 2

**DOI:** 10.5256/f1000research.176032.r399331

**Published:** 2025-07-28

**Authors:** Abayeneh Girma

**Affiliations:** 1 Mekdela Amba University, Tulu Awlya, South Wollo Zone, Ethiopia

**General comments**

 The manuscript addresses an important topic—prescription patterns and adverse drug reactions (ADRs) in urinary tract infections (UTIs) among reproductive-age women. However, significant methodological and conceptual flaws persist despite revisions, as highlighted by previous reviewers. The study’s design, sample size justification, and clarity of objectives remain inadequate, compromising its scientific rigor and reproducibility.

**Major** **comments**

1. **Sample size and statistical power**: The sample size calculation (n=47) is based on UTI prevalence (3.14%) rather than ADR prevalence (typically 5–15%), as correctly noted by Reviewer 1. This undermines the study’s ability to detect meaningful ADR patterns. A sample size of 47 is insufficient for robust conclusions, especially for secondary outcomes like combination therapy analysis.
2. **Study design and methodology** (i) **Retrospective vs. prospective contradictions:** The authors claim a retrospective design but propose telephonic follow-ups for ADR data, which introduces prospective elements. This hybrid approach lacks justification and risks bias (e.g., recall bias, non-response bias). (ii) **Data source clarity:** The manuscript does not specify whether UTIs are community-acquired or hospital-acquired, nor does it define diagnostic criteria (e.g., symptoms, culture results). This omission limits the interpretability of prescription patterns. (iii) **ADR collection:** Relying on hospital records for ADRs is problematic, as ADRs are often underreported in retrospective data. The plan to contact patients by phone is not standardized or ethically clarified (e.g., consent process).
3. **Objectives and scope**: The primary objectives are clear but overly broad. For example, "analyzing adverse reactions" lacks specificity (e.g., which ADRs, severity grading). Again, the secondary objective to analyze fixed-dose combinations (FDCs) is commendable but poorly operationalized. Examples like "Nitrofurantoin + Vitamin B6" are questionable, as Vitamin B6 is not a standard UTI treatment.
4. **Peer review responses**: The authors’ responses to reviewers (e.g., sample size recalculation, ADR methodology) are superficial. Key critiques (e.g., hospital vs. community UTI distinction, statistical methods) remain unaddressed.
5. **Ethical and practical issues**: While ethical approval is mentioned, the waiver for patient consent in retrospective data collection is noted. However, telephonic follow-ups for ADRs would require explicit ethical approval and consent, which is not discussed.

**Minor comments**

1. **Introduction/Discussion:** Redundant and lacks critical synthesis of prior literature (e.g., no mention of regional antibiotic resistance trends in Central India).
2. **Table 1 (Data Collection):** Parameters are listed but lack operational definitions (e.g., "irrational prescription" criteria).
3. **Statistical analysis:** Mention of "Student’s t-test and Chi-square" is insufficient. No details on covariates, regression models, or handling missing data.

Is the study design appropriate for the research question?

No

Is the rationale for, and objectives of, the study clearly described?

No

Are sufficient details of the methods provided to allow replication by others?

No

Are the datasets clearly presented in a useable and accessible format?

No

Reviewer Expertise:

Microbiology

I confirm that I have read this submission and believe that I have an appropriate level of expertise to state that I do not consider it to be of an acceptable scientific standard, for reasons outlined above.

# Response

**Published:** 2025-07-31

**Authors:** DR. GAYATRI PATIL

**Affiliations:** PHARMACOLGY, Not Applicable, WARDHA, MAHARASHTRA, India

**Response to Reviewer: Dr. Niels Frimodt-Møller**

**COMMENT 1:**

 “The calculation of sample size is not correct; With a prevalence of around 3% (and what does that mean?) of UTIs in India they propose, that 47 patients will suffice? The prevalence of adverse effects is probably 10-15%; the investigators will need at least 10 times as many patients to achieve a reasonable result.”

**AUTHOR RESPONSE:**

 We thank the reviewer for this important observation. We agree that calculating sample size based on UTI prevalence was not appropriate for our primary objective, which includes the analysis of adverse drug reactions (ADRs).

 In response, we have **recalculated the sample size** using Daniel’s formula, based on an **assumed ADR prevalence of 10%**, with 95% confidence level and a 5% margin of error.

**Formula used:**

 n = Z² × P × (1 − P) / d²

 Z = 1.96, P = 0.10, d = 0.05

 n = (1.96)² × 0.10 × (0.90) / (0.05)² ≈ 138.3

 We have therefore revised the sample size to **139 patients** and updated this in the **Sample Size Estimation** section. This revised sample size is now aligned with our primary outcome — the analysis of adverse drug reactions. We used an assumed ADR prevalence of 10%, as reported in similar studies (e.g., Fatima et al., J Drug Deliv Ther, 2015). This reference has been cited in the updated manuscript to support our methodology.

**COMMENT 2:**

 The method to study adverse effects is to look in the charts (response to reviewer 1), while they also want to call by phone the patients in question (response to reviewer 2); in the last case it is not a retrospective study. Which charts are we talking about? If hospital charts, then the patients belong to a special group of patients admitted to hospital probably with complicated UTI, where adverse effects are different from community dwellers with uncomplicated UTI.

**AUTHOR RESPONSE:**

 Thank you for pointing out this inconsistency. We have now removed the telephonic follow-up plan and clarified that the study will be conducted as a purely retrospective observational study. Only medical records from the hospital’s archived data will be used.

 The protocol has been updated to reflect this in the Study Design, Data Collection, and Ethical Considerations sections. We will rely solely on documented ADRs recorded in the patient files.

 This revision ensures methodological consistency with retrospective design principles and aligns with the ethical waiver granted by our Institutional Ethics Committee.

 We have now specified that our data will be collected only from outpatient department (OPD) records of women diagnosed and treated for UTI. This ensures that our focus is on community-acquired, uncomplicated UTIs.

 This clarification is now included in the Study Setting, Inclusion Criteria, and Data Collection sections. No inpatient or ICU cases will be included, and complicated UTI cases will be excluded.

**COMMENT 3:**

 The introduction has not been revised as proposed by reviewer 1, and neither the discussion as also recommended.

**AUTHOR RESPONSE:**

 We thank the reviewer for this feedback. We have now extensively revised both the Introduction and Discussion sections.

 The Introduction now includes recent literature on UTI treatment patterns, local prescribing practices, and rationale for ADR monitoring in reproductive-age women.

 The Discussion has been expanded to address implications of irrational prescribing, potential underreporting of ADRs, and the regional significance of our findings. We have also added a paragraph on study limitations to improve transparency.

 All changes are clearly marked in the revised manuscript.

**COMMENT 4:**

 The different treatments of UTI described in the protocol are mostly for uncomplicated UTI, which does not comply with the idea, that hospital charts are being scrutinized.

**AUTHOR RESPONSE:**

 As noted above, we have now explicitly stated that the study will include only outpatient records, and our focus is on community-acquired uncomplicated UTIs.

 Accordingly, the treatment guidelines referenced in the manuscript pertain to uncomplicated UTI management, and this is now clearly stated in the revised Treatment and Study Design sections.

# Reviewer report: Reviewer response for version 2

**DOI:** 10.5256/f1000research.176032.r365731

**Published:** 2025-02-13

**Authors:** Niels Frimodt-Møller

**Affiliations:** 1 Copenhagen University Hospital, Copenhagen, Denmark

The authors' responses to the reviewers' suggestions seem a bit unclear, and it appears that my original comments may not have been fully addressed.

- The calculation of sample size is not correct; With a prevalence of around 3% (and what does that mean?) of UTIs in India they propose, that 47 patients will suffice? The prevalence of adverse effects is probably 10-15 %; the investigators will need at least 10 times as many patients to achieve a reasonable results.
- The method to study adverse effects is to look in  the charts (response to reviewer 1), while they also want to call by phone the patients in question (response to reviewer 2); in the last case it is not a retrospective study. Which charts are we talking about? If hospital charts, then the patients belong to a special group of patients admitted to hospital probably with complicated UTI, where adverse effects are different from community dwellers with uncomplicated UTI.
- The introduction has not been revised as proposed by reviewer 1, and neither the discussion as also recommended.
- The different treatments of UTI described in the protocol are mostly for uncomplicated UTI, which does not comply with the idea, that hospital charts are being scrutinized.
- The authors need to respond to the comments and completely revise the protocol.

Is the study design appropriate for the research question?

No

Is the rationale for, and objectives of, the study clearly described?

No

Are sufficient details of the methods provided to allow replication by others?

No

Are the datasets clearly presented in a useable and accessible format?

No

Reviewer Expertise:

Clinical microbiology, urinary tract infections, E.coli pathogenesis, antibiotic activity in vitro and in vivo

I confirm that I have read this submission and believe that I have an appropriate level of expertise to state that I do not consider it to be of an acceptable scientific standard, for reasons outlined above.

# Reviewer report: Reviewer response for version 1

**DOI:** 10.5256/f1000research.163709.r297784

**Published:** 2024-07-13

**Authors:** Vineetha Menon

**Affiliations:** 1 Gulf Medical University, Ajman, United Arab Emirates

1. Abstract: The abstract is too brief; lacks specificity and content. 

 2. Introduction: The introduction lacks smooth flow and transitions between sections. There is some redundancy of information, grammatical errors, and formatting issues. There is no information given on the findings from previous studies done in this area. Also, there is a lack of focus on the significance of the study. 

 3. Method:

 a. Study criteria: Study criteria is missing out on several vital elements. For example, details of treatment settings, time frame, presence of comorbidities, pregnancy status, or recurrent infections had not been addressed in the study criteria. 

 b. What does the author mean by " **Study reference**: Pritam Pardeshi"?

 c. Statistical methods: There is a lack of information on how statistical methods were applied.

 d. Dala collection: The data collection process is not detailed enough. 

 e. Study setting: The study setting does not mention in which institution the study was done. 

 f. Overall there are a lot of inconsistencies in the methodology. It's also stating that the study will be done- what does that mean?

 4. Results: No results are available for review. 

 5. Discussion: The discussion lacks depth and specificity. There is also repetition of information previously mentioned. No clear conclusion or limitations were stated.

Is the study design appropriate for the research question?

Yes

Is the rationale for, and objectives of, the study clearly described?

No

Are sufficient details of the methods provided to allow replication by others?

No

Are the datasets clearly presented in a useable and accessible format?

No

Reviewer Expertise:

Geriatrics, Medication safety management

I confirm that I have read this submission and believe that I have an appropriate level of expertise to state that I do not consider it to be of an acceptable scientific standard, for reasons outlined above.

## References

[1] : Study on Urinary Tract Infection Among Females of Reproductive Age Group in Tertiary Care Teaching Hospital, Dhaka, Bangladesh. *European Journal of Medical and Health Sciences* .2021;3(1) : 10.24018/ejmed.2021.3.1.680 85-89 10.24018/ejmed.2021.3.1.680

[2] : Antimicrobial prescribing pattern in urinary tract infection in a tertiary care hospital. *National Journal of Physiology, Pharmacy and Pharmacology* .2017; 10.5455/njppp.2017.7.0623506072017 10.5455/njppp.2017.7.0623506072017

[3] : Prescription pattern of antibiotic usage for urinary tract infection in tertiary care hospital. *World Journal of Biology Pharmacy and Health Sciences* .2023;16(1) : 10.30574/wjbphs.2023.16.1.0423 178-181 10.30574/wjbphs.2023.16.1.0423

# Response

**Published:** 2024-12-16

**Authors:** DR. GAYATRI PATIL

**Affiliations:** PHARMACOLGY, Not Applicable, WARDHA, MAHARASHTRA, India

**1. Abstract:** The abstract is too brief; lacks specificity and content. 

**Response:**    Changes done in the abstract.

**2. Introduction:** The introduction lacks smooth flow and transitions between sections. There is some redundancy of information, grammatical errors, and formatting issues. There is no information given on the findings from previous studies done in this area. Also, there is a lack of focus on the significance of the study. 

**Response:**   Now, I have mentioned the information regarding the previous study. It is mentioned in the discussion part. i.e.

 This analysis is for the use of evidence-based medicine in tertiary care centers, to fulfill the drug information needs of the physician and also for improvement in the quality of healthcare by giving feedback to prescribers. This is an observational, retrospective study that will aim at the analysis of drug prescription patterns and adverse drug reactions due to drug prescription in urinary tract infections at tertiary health care centers.

**3. Method:**

 a. Study criteria: Study criteria is missing out on several vital elements. For example, details of treatment settings, time frame, presence of comorbidities, pregnancy status, or recurrent infections had not been addressed in the study criteria.

**Response:** I have now added these parameters in my annexure

 NAME, AGE/SEX, MRD NO., OPD NO., DIAGNOSIS, DRUGS USED, DOSE OF THE DRUG, ANY IRRATIONAL IN PRESCRIPTION, DURATION OF DRUG THERAPY, ADVERSE DRUG REACTIONS, DURATION OF ILLNESS, COMORBIDITIES, PREGNANCY STATUS, RECURRENT INFECTIONS

**b. What does the author mean by "Study reference: Pritam Pardeshi"?**

**Response:** This was the reference for my sample size calculation for the study.

**c. Statistical methods: There is a lack of information on how statistical methods were applied.**

**Response:** We will use the student’s t-test and Chi-square Test.

**d. Data collection: The data collection process is not detailed enough.**

**Response**: I will be collecting the data from the medical record section and then I will analyze the prescription for the given parameters which were given in the data collection section in my protocol.

**e. Study setting: The study setting does not mention in which institution the study was done**. 

**Response:  **This study will be carried out in the Rural Tertiary Care Hospital in Central India

**f. Overall there are a lot of inconsistencies in the methodology. It's also stating that the study will be done- what does that mean?**

**Response:  **We haven’t started this study yet that’s why I wrote the study will be done. It’s my protocol for my study.

**4. Results: No results are available for review.**

**Response:  **As this study is not started yet we can’t predict the result of this analysis. That’s why I didn’t mention the results.

**5. Discussion: The discussion lacks depth and specificity. There is also a repetition of information previously mentioned. No clear conclusion or limitations were stated.**

**Response:  **I have made the changes in the discussion part according to your comments.

  

- **Is the rationale for, and objectives of, the study clearly described?**

No, now I have made the changes to the objective.

- **Is the study design appropriate for the research question?**

Yes

- **Are sufficient details of the methods provided to allow replication by others?**

No **Response:  ** Changes were made in the data collection section.

- **Are the datasets clearly presented in a useable and accessible format?**

No

**Response:  **  I have mentioned the parameters in this ANNEXURE.

 NAME, AGE/SEX, MRD NO., OPD NO., DIAGNOSIS, DRUGS USED, DOSE OF THE DRUG, ANY IRRATIONAL IN PRESCRIPTION, DURATION OF DRUG THERAPY, ADVERSE DRUG REACTIONS, DURATION OF ILLNESS, COMORBIDITIES, PREGNANCY STATUS, RECURRENT INFECTIONS

# Reviewer report: Reviewer response for version 1

**DOI:** 10.5256/f1000research.163709.r276370

**Published:** 2024-05-28

**Authors:** Niels Frimodt-Møller

**Affiliations:** 1 Copenhagen University Hospital, Copenhagen, Denmark

The protocol by Patil et al concerns an interesting subject regarding antibiotic use in urinary tract infection (UTI) in women, but the protocol needs revision particluarly to be more strict and prescise.

 1. It is not clear, whether the investigation partakes on nosocomial (hospital derived) UTI or community associated UTI, or both? Define.

 2. Define how a UTI is going to be defined through the retrospective approach (symptoms, urine culture, biochemical issue etc), if it is going to be analysed, whether the antibiotic treatment was correct i.e. administered for UTI.

 3. Define which adverse effects are searched for, not only for nitrofurantoin and sulfonamides? it is often the case, that hospital records do not monitor adverse effects - this is always better done in a prospective study, where such effects are defined and the investigators asked to record them.

 4. The calculation of sample size is performed according to prevalence of UTI; since all subjects searched for are supposed to have UTI, this is not correct. The sample size should be based upon the theoretical prevalence of adveres effects, since this is main objective. This is usually in the range of 5-10%, why the sample size must be much larger.

 5. What is meant by combination therapy and fixed combinations? describe which e.g. trimethoprim-sulphamethoxazole, amoxicilin-clavulanic acid etc. Is combination therapy meant to be two drugs both intended for UTI?

Is the study design appropriate for the research question?

No

Is the rationale for, and objectives of, the study clearly described?

No

Are sufficient details of the methods provided to allow replication by others?

No

Are the datasets clearly presented in a useable and accessible format?

No

Reviewer Expertise:

Clinical microbiology, urinary tract infections, E.coli pathogenesis, antibiotic activity in vitro and in vivo

I confirm that I have read this submission and believe that I have an appropriate level of expertise to state that I do not consider it to be of an acceptable scientific standard, for reasons outlined above.

# Response

**Published:** 2024-12-16

**Authors:** DR. GAYATRI PATIL

**Affiliations:** PHARMACOLGY, Not Applicable, WARDHA, MAHARASHTRA, India

**1. It is not clear, whether the investigation partakes on nosocomial (hospital derived) UTI or community associated UTI, or both? Define.**

**RESPONSE:** This study only focuses on prescription patterns so this study has no relationship with investigations.

**2. Define how a UTI is going to be defined through the retrospective approach (symptoms, urine culture, biochemical issue etc), if it is going to be analysed, whether the antibiotic treatment was correct i.e. administered for UTI.**

**RESPONSE: ** This study has no relation with symptoms, urine culture, or biochemical issues this study will focus only on the treatment part after analyzing we will see the pattern of prescription and adverse drug reactions of drugs with each prescription. So focusing on the treatment part, we can’t define UTI.

**3. Define which adverse effects are searched for, not only for nitrofurantoin and sulfonamides? it is often the case, that hospital records do not monitor adverse effects - this is always better done in a prospective study, where such effects are defined and the investigators asked to record them.**

**RESPONSE : **Although it is a retrospective study, to analyze the ADR aspect we are going to telephonically contact them and get the feedback.

**4. The calculation of sample size is performed according to prevalence of UTI; since all subjects searched for are supposed to have UTI, this is not correct. The sample size should be based upon the theoretical prevalence of adveres effects, since this is main objective. This is usually in the range of 5-10%, why the sample size must be much larger.**

**RESPONSE:** Changes done in sample size **.**

**5. What is meant by combination therapy and fixed combinations? describe which e.g. trimethoprim-sulphamethoxazole, amoxicilin-clavulanic acid etc. Is combination therapy meant to be two drugs both intended for UTI?**

**RESPONSE:**

**Fixed-dose drug combination (FDC**) refers to a pharmaceutical formulation that contains two or more active ingredients in a single dosage form.

 Ex. Trimethoprim + Sulfamethoxazole (Co-trimoxazole)

      Amoxicillin + Clavulanic Acid

      Nitrofurantoin + Vitamin B6

**Combination therapy**: For ex. If there are 6 different drugs which are available for the treatment of UTI. The combination of six different drug is combination therapy.

 Hence both the terminologies are different.

**Is the rationale for, and objectives of, the study clearly described?**

 No **RESPONSE:** I have made the change in objective.

**Is the study design appropriate for the research question?**

 No 

**RESPONSE:  **Although it is a retrospective study, to analyze the ADR aspect we are going to telephonically contact them and get the feedback.   I am doing the retrospective study as this is the lacunae of the institute that’s why I am getting the information on ADR by communicating with the same patients.

- **Are sufficient details of the methods provided to allow replication by others?**

No **RESPONSE:** Changes were made in the data collection section.

  

- **Are the datasets clearly presented in a useable and accessible format?**

No **RESPONSE :** I have mentioned the parameters in the annexure.

 NAME, AGE/SEX, MRD NO., OPD NO., DIAGNOSIS, DRUGS USED, DOSE OF THE DRUG, ANY IRRATIONAL IN PRESCRIPTION, DURATION OF DRUG THERAPY, ADVERSE DRUG REACTIONS, DURATION OF ILLNESS, COMORBIDITIES, PREGNANCY STATUS, RECURRENT INFECTIONS
